# Production of (10*S*,11*S*)-(—)-*epi*-Pyriculol and Its HPLC Quantification in Liquid Cultures of *Pyricularia grisea*, a Potential Mycoherbicide for the Control of Buffelgrass (*Cenchrus ciliaris*)

**DOI:** 10.3390/jof9030316

**Published:** 2023-03-03

**Authors:** Jesús G. Zorrilla, Marco Masi, Suzette Clement, Alessio Cimmino, Susan Meyer

**Affiliations:** 1 Department of Chemical Sciences, University of Naples Federico II, Complesso Universitario Monte S. Angelo, Via Cintia, 80126 Naples, Italy; 2 Allelopathy Group, Department of Organic Chemistry, Facultad de Ciencias, Institute of Biomolecules (INBIO), University of Cadiz, C/Avenida República Saharaui, s/n, 11510 Puerto Real, Spain; 3 Shrub Sciences Laboratory, U.S. Forest Service Rocky Mountain Research Station, 369 N. 100 West Suite 8, Cedar City, UT 84721, USA

**Keywords:** (10*S*,11*S*)-(—)-*epi*-pyriculol, HPLC method, qualitative and quantitative analysis, phytochemistry, natural products

## Abstract

(10*S*,11*S*)-(—)-*epi*-pyriculol is a phytotoxic metabolite produced by *Pyricularia grisea*, a fungus identified as a foliar pathogen on the invasive weed species buffelgrass (*Cenchrus ciliaris*) in North America. The effective control of buffelgrass has not yet been achieved, and there is a need to develop effective and green solutions. Herbicides based on natural products and the use of phytopathogenic organisms could provide the most suitable tools for the control of weeds such as buffelgrass. Thus, one of the most relevant points to study about potential suitable phytotoxins such as (10*S*,11*S*)-(—)-*epi*-pyriculol is its production on a large scale, either by isolation from fungal fermentations or by synthesis. For these purposes, rapid and sensitive methods for the quantification of (10*S*,11*S*)-(—)-*epi*-pyriculol in complex mixtures are required. In this study, a high-pressure liquid chromatography (HPLC) method for its quantification was developed and applied to organic extracts from twelve *P. grisea* isolates obtained from diseased buffelgrass leaves and grown in potato dextrose broth (PDB) liquid cultures. The analysis proved that the production of (10*S*,11*S*)-(—)-*epi*-pyriculol is fungal-isolate dependent and strongly correlated with phytotoxic activity, shown by the *P. grisea* organic extracts in a buffelgrass radicle elongation test. The HPLC method reported herein allowed us to select the best strain for the production of (10*S*,11*S*)-(—)-*epi*-pyriculol and could be useful for selecting the best cultural conditions for its mass production, providing a tool for the use of this promising metabolite as a new bioherbicide for the control of buffelgrass.

## 1. Introduction

**Figure 1:**
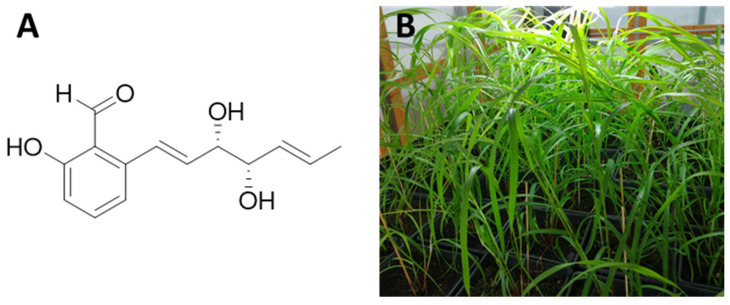
(**A**) Structure of (10*S*,11*S*)-(—)-*epi*-pyriculol (EPYR); (**B**) greenhouse-grown *Cenchrus ciliaris* (buffelgrass).

The study of the metabolites produced by phytopathogenic fungi is a suitable tool for broadening the current knowledge of allelopathy between species in ecosystems, as well as for discovering useful, natural products that could be employed for the development of new herbicides [1]. Allelochemicals are, in fact, considered natural herbicides, so using them as models for the development of herbicides can lead to a series of unique advantages [2]. Compounds produced by phytopathogenic fungi are of great interest in this context, since they are often related to the development of plant diseases (with specific symptoms such as wilting, suppression of growth, chlorosis, necrosis, or leaf spots), being mostly low-molecular-weight secondary metabolites [3]. Fungal species belonging to genera such as *Botrytis*, *Pyrenophora*, or *Pyricularia* have been discovered as producers of phytotoxic metabolites with potential use in agriculture [1,4,5,6]. Specifically, the genus *Pyricularia* has been a focus of attention. This has led to the identification of diverse phytotoxins such as pyriculariol and some of its isomers, pyrichalasin H, terrestic acid, pyriculins A and B, and some napthalenones, among others [7,8,9,10,11,12]. Some of these studies proved that (10*S*,11*S*)-(—)-*epi*-pyriculol (Figure 1A, hereinafter abbreviated to “EPYR”), isolated from *Pyricularia oryzae* or *Pyricularia grisea*, is a metabolite with potent phytotoxic activity against rice and the weed buffelgrass (*Cenchrus ciliaris*) [9,11] (Figure 1B).

The interest in further research on EPYR stems from its phytotoxic activity against buffelgrass, an invasive weed, the expansion of which, compounded by a capacity for rapid invasion and a high tolerance to drought, threatens ecosystems worldwide [13]. The available methods for the control of buffelgrass are still not target specific and, thus, have negative impacts, and the use of phytotoxins produced by weed-pathogenic fungi has been considered as a potential solution for the management of this weed [14]. When bioassayed in a buffelgrass coleoptile and radicle elongation test at a concentration of 5 × 10^−3^ M, EPYR showed toxicity, reducing seed germination with respect to the control and inhibiting the elongation of buffelgrass radicles [11]. Furthermore, when tested by leaf puncture assay on buffelgrass leaves, EPYR showed strong necrosis at 2.5 × 10^−3^ M and was also active at a concentration of 10^−3^ M [15]. Thus, there is an interest in improving production of EPYR to allow new, larger-scale studies, ideally under greenhouse and field conditions. The availability of good quantities of EPYR will also allow us to perform ecotoxicological studies using organisms representing different trophic levels of aquatic and terrestrial ecosystems, namely, producers (e.g., the green freshwater alga *Raphidocelis subcapitata* and the macrophyte *Lepidium sativum*), consumers (e.g., the water flea *Daphnia magna* and nematode *Caenorhabditis elegans*), and decomposers (e.g., the bacterium *Aliivibrio fischeri*) [16]. The development of rapid and sensitive methods for the quantification of EPYR in complex mixtures is needed for these purposes, as are the requirements for modern analysis, identification, and quantification of compounds in natural samples in general [17,18]. The quantification of specific bioactive compounds produced by microorganisms and plants is, in fact, a focus of attention in allelopathy studies [19], and, serving as a clear example, is the quantification study of the phytotoxin tenuazonic acid produced by *P. oryzae* [20].

This study reports the development of a suitable method, based on high-pressure liquid chromatography (HPLC), for qualitative and quantitative rapid analysis of fungal samples containing EPYR. Particularly, the organic extracts obtained from the liquid culture (potato dextrose broth, PDB) of twelve isolates of *P. grisea* from diseased buffelgrass leaves in North America were evaluated for their content of EPYR. Evaluation of the phytotoxicity of *P. grisea* isolate extracts on buffelgrass seedlings was performed, making it possible to correlate the phytotoxic activity level with the amount of EPYR contained in the organic extracts of the different isolates.

## 2. Materials and Methods

### 2.1. General Experimental Procedures

Analytical thin-layer chromatography (TLC) was performed on silica gel plates (Kieselgel 60 F_254_, 0.25 mm, Merck, Darmstadt, Germany). Spots were visualized by exposure to UV radiation (254 nm) and by spraying with 10% H_2_SO_4_ in methanol (MeOH) (*v/v*) and then with a 5% solution of phosphomolybdic acid in ethanol (*v/v*), followed by heating at 110 °C for 10 min. Sigma-Aldrich Co. (St. Louis, MO, USA) supplied all solvents. Optical rotations were measured in chloroform (CHCl_3_) on a JASCO (Tokyo, Japan) P-1010 digital polarimeter. ^1^H NMR spectra were recorded in deuterated chloroform (CDCl_3_) at 400 MHz on a Bruker (Karlsruhe, Germany) spectrometer, and CDCl_3_ was used as an internal standard. Electrospray ionization mass spectra (ESIMS) were recorded on a LC/MS TOF apparatus Agilent 6230B (Agilent Technologies, Milan, Italy).

### 2.2. Pyricularia grisea Isolates

Twelve isolates of *P. grisea* were obtained from diseased buffelgrass tissue collected in southern Hidalgo County, TX, USA (isolates 1–3), and Saguaro National Monument, AZ, USA (isolates 4–12). Positive identification for each isolate was obtained using Sanger sequencing of the standard ITS bar code region for fungi [21] followed by a blast search in the Genbank database. All the isolates were maintained on potato dextrose agar (PDA, Fluka, Sigma-Aldrich Chemic GmbH, Buchs, Switzerland) and stored at 4 °C in the strain collection of S. Meyer at the USFS RMRS Shrub Sciences Laboratory, Provo, UT, USA.

### 2.3. Production and Extraction of Liquid Cultures

To obtain the culture filtrates used in this study, the twelve isolates (isolates 1–12) were grown in PDB at room temperature by inoculation of sterile broth (125 mL) in 1 L Erlenmeyer flasks containing fragments of mycelial mat produced on potato dextrose agar (PDA). After inoculation, they were incubated in shaker culture for 14 days and then centrifuged and filtrated to remove the residual mycelium. Once their pH values were measured, the resulting filtrates were lyophilized and stored at −20 °C. To obtain the extracts, the lyophilized culture filtrates were dissolved in 20 mL of distilled H_2_O and extracted with ethyl acetate (EtOAc) (3 × 25 mL). The organic extracts were combined, dried over anhydrous Na_2_SO_4_, and evaporated under reduced pressure to obtain brown oily residues (extracts 1–12). The residue aqueous phase of each filtrate was then acidified to pH 2 by addition of formic acid and extracted with EtOAc (3 × 25 mL) to obtain twelve new extracts (following the same procedure.

### 2.4. Isolation and Identification of (10S,11S)-(—)-epi-Pyriculol

(10*S*,11*S*)-(—)-*epi*-Pyriculol (EPYR) (Figure 1A), used in this study as the standard for the HPLC analysis, was isolated from in vitro PDB cultures of *P. grisea* SNM22 according to previously published procedures [11]. Its purity was verified by means of HPLC, NMR, and mass spectrometry. The experimental ^1^H NMR data, provided in the Supporting Information (S1), were in agreement with those reported in literature for (10*S*,11*S*)-(—)-*epi*-pyriculol, as well as the peaks observed in the ESI MS spectrum (see Figure S2) and its specific optical rotation value ([α]^25^_D_ = −31° in CHCl_3_ at *c* 0.40; lit. value = −31° in CHCl_3_ at *c* 0.41) [9,11]. Following the same isolation procedures, EPYR was also obtained and spectroscopically characterized from aliquots of the richest cultures in EPYR (SNM 5A2 and SNM 2–1A3) to corroborate its presence in the samples under study.

### 2.5. HPLC System and Quantification of (10S,11S)-(—)-epi-Pyriculol

The calibration of a curve for the quantification of EPYR, and its quantification in the *P. grisea* extracts, was performed using a Hitachi HPLC system paired with a 5160 pump and a 5410 spectrophotometric detector. A Phenomenex C18 reversed-phase column Lichrocart (250 × 4.6 mm i.d.; 5 µm) was used for the separations and a mixture of MeOH–H_2_O (1% formic acid) as mobile phase at a flow rate of 0.5 mL/min. The analyses were performed by using a MeOH–H_2_O (1% formic acid) gradient from 50% MeOH to 70% in a linear increment for 30 min and, finally, linearly re-balanced to the initial rate for 10 min. All analyses were monitored for 50 min. Samples, previously filtered through 0.45 μm filters, were accurately dissolved in MeOH at 2 mg/mL and injected through a 20 μL loop. The analysis of each sample was performed in triplicate. The standard sample of EPYR was employed for the optimization and calibration of the curve by its injection in a range of 0.0078–5 μg. The detection of EPYR was performed by displaying a wavelength value of 231 nm, in accordance with the maximum UV absorbance peak reported in the literature [9].

### 2.6. Coleoptile and Radicle Elongation Bioassay

The phytotoxicity of the organic extracts obtained from the twelve *P. grisea* isolates (1–12), obtained at regular pH and pH 2, was tested in coleoptile and radicle elongation bioassays against buffelgrass (*C. ciliaris*) following the procedure reported by Masi et al. [11]. Briefly, the extracts were dissolved in dimethyl sulfoxide (DMSO) and brought up to the assay concentration of 2 mg/mL with distilled water (final DMSO concentration of 2%, *v/v*). For each sample, 250 µL of the solution was applied into three 3.5 cm Petri dishes onto the surface of filter paper. A solution of 5 µL of DMSO in 245 µL of H_2_O was used as negative control. Three replicates were performed for each sample and the negative control. Four buffelgrass seeds were then placed onto the surface of each filter paper, and the Petri dishes were sealed with parafilm and incubated at 20 °C in an incubator with a light–darkness photoperiod of 12–12 h. The germination was scored daily and recorded for each seed, and its radicle and coleoptile length were measured and recorded 3 days after germination using an electronic caliper. Those seeds that did not germinate after 7 days (<5%) were excluded from the analysis. For samples with seeds that showed radicle growth but did not develop a coleoptile, coleoptile length was scored as zero. Seeds that exhibited coleorhiza extrusion but no radicle or coleoptile elongation were scored as germinated but with both radicle and coleoptile lengths of zero. Seedling growth data were analyzed on a per-seed basis across all replicates for each treatment.

## 3. Results and Discussion

Twelve isolates (1–12) of *P. grisea* were grown in PDB, and their culture filtrates were extracted following the procedures described in the Materials and Methods section. After completion of the extraction at normal pH, it should be noted that all the filtrates were extracted again after acidifying to pH 2, given the relevance of the pH in the biosynthesis of fungal metabolites [22]. Data about the isolates, their culture filtrates, and organic extract yields obtained from 125 mL of PDB culture are summarized in Table 1.

The weights of the lyophilized culture filtrate residues of the twelve isolates were comparable, as were those of the corresponding mycelia. However, isolates 8 and 10 (SNM 3E2A and SNM 5B1, respectively) showed higher amounts of lyophilized culture filtrate and a lower yield of mycelia, suggesting their poor affinity for the PDB liquid culture in comparison with the other isolates (1–7, 9, 11, or 12). Most culture filtrates had a basic pH except for isolates 5, 8, and 10 (mild acid pH) and isolate 11 (neutral pH), indicating the release of structurally diverse compounds, the mixture of which was able to modify the regular pH of media (pH 7). It is worth noting the adaptive capacity of fungi to pH changes, being able to modulate the pH of their environment by secreting acid or basic metabolites, as described for a wide number of species such as *P. grisea* [23]. Regarding the results obtained by carrying out the extraction with EtOAc at regular pH of the culture filtrates, isolates 4 and 9 showed the best yield with values (mg) that were almost double those obtained with the other isolates. Finally, most of the organic extracts obtained at pH 2 showed a significantly higher yield than those at regular pH, with the exception of isolates 1 and 7, obtained in similar yields (Table 1). A TLC analysis carried out using a mixture of CHCl_3_-*iso*-propanol (9:1, *v/v*) and EPYR as a standard sample revealed that the organic extracts (obtained at both pHs) contained variable amounts of this compound. In particular, this qualitative analysis indicated that the organic extracts obtained from isolates 3, 4, and 9 contained the highest amount of EPYR among the regular and pH 2 extracts. To confirm these preliminary results, the quantification of EPYR (Figure 1) in the different organic extracts of isolates 1–12 was performed. A HPLC method was developed, applying the methodology detailed in the Materials and Methods section. The validation of this HPLC method was in agreement with the rules reported in the “Guidance for Industry—Bioanalytical Method Validation” published by the Food and Drug Administration (FDA, White Oak, MD, USA) [24,25] in terms of limit of quantitation (LOQ), intra- and inter-assay precision, and accuracy. Data from analyses of EPYR standard dissolved in MeOH (absolute amount vs. chromatographic peak area), following the reported method, allowed the calculation of a suitable linear regression curve with the characteristics summarized in Table 2. A graphical representation of the calibration curve is provided in the Supporting Information.

**Figure 2:**
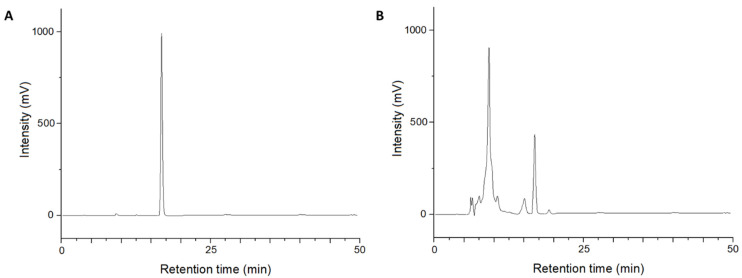
(**A**) Chromatogram of EPYR standard analyzed at 250 μg/mL; (**B**) chromatogram of the organic extract obtained at regular pH of isolate 9 (SNM 5A2), analyzed at 2 mg/mL.

Retention times were highly reproducible, with variations below 0.12 min. The limit of detection (LOD) was extrapolated from the calibration curve according to the IUPAC guidelines [26]. The chromatographic profile of EPYR at 250 μg/mL is shown in the chromatogram depicted in Figure 2A.

The quantitative determination of EPYR in the EtOAc organic extracts obtained from the twelve *P. grisea* isolates (1–12) was calculated by interpolating the mean area of the chromatographic peaks using the equation from the calibration curve (Table 2). The relative amounts (% *w/w*) of EPYR in the organic extracts of isolates 1–12 obtained at regular pH and pH 2, as well as the production yields (mg/L) in PDB liquid culture, are shown in Table 3.

This quantitative analysis validated the preliminary results obtained, evaluating the TLC profile of the organic extracts of the twelve isolates. In fact, isolates 3, 4, and 9 showed the highest percentage of EPYR in the organic extract, obtained at regular pH, as well as the best production yield. In particular, isolate 9 (SNM 5A2) was the best producer of EPYR with a production yield of 10.46 ± 0.21 mg/L (Figure 2B). The other isolates showed low production of EPYR, and, for some of them (namely, isolates 8 and 12), the production of EPYR was probably below the limit of detection for the developed method. The same trend was also observed for the organic extracts obtained at pH 2. In particular, the compound was, again, detected primarily in extracts from isolates 4 and 9 but in lesser quantities than from the regular pH extractions.

**Figure 3:**
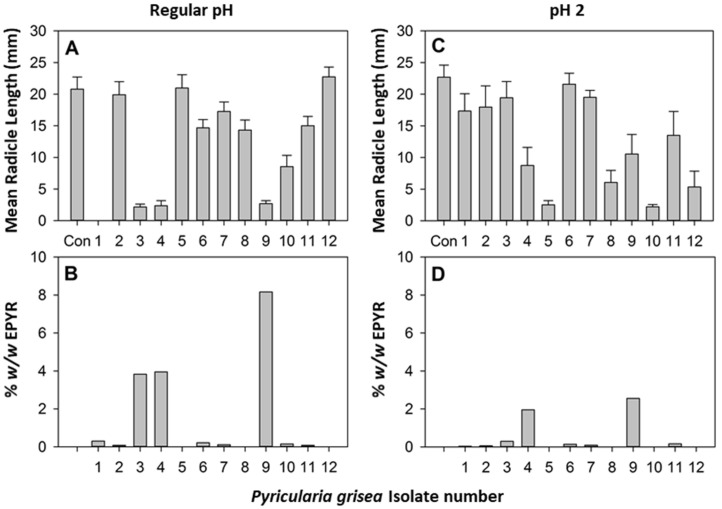
Effect of *Pyricularia grisea* organic extraction at regular pH from culture filtrates of the twelve *P. grisea* isolates (1–12) on (**A**) 3-day radicle length in a buffelgrass seedling bioassay and (**B**) EPYR concentrations in those 12 extracts. (**C**,**D**) Parallel results from organic extraction at pH 2. Error bars represent standard error of the mean.

To understand if there is a relationship between the EPYR production and phytotoxicity of the twelve isolates of *P. grisea*, their organic extracts were tested in a buffelgrass coleoptile and radicle elongation test following the procedure described in the Materials and Methods section. The results are reported in Figure 3.

There was no significant effect of isolate, extraction pH, or their interaction on coleoptile elongation (data not shown). In contrast, there were large and significant differences among isolates in their phytotoxic effect on buffelgrass radicle elongation and also a significant interaction as a function of pH (isolate: d.f. = 12, 93, F = 10.16, *p* < 0001; isolate x pH: d.f. = 11, 93, F = 7.21, *p* < 0.0001). The organic extracts obtained at regular pH from isolates 3, 4, and 9 were very phytotoxic, reducing the radicle development of buffelgrass seedlings by almost 90% with respect to the control (Figure 3A); these were the three extracts that contained by far the highest concentrations of EPYR (Figure 3B). This result demonstrated the strong relationship between the phytotoxicity of the isolates and production of EPYR. However, the presence of other compounds in the organic extracts, as well as their synergism, could also play a role in their phytotoxic activity, as reported for other examples of phytotoxic fungal compounds [27]. The synergistic effect of pyrenophoric acid and cytochalasin B, two fungal metabolites produced by *Pyrenophora semeniperda* and belonging to different classes of natural compounds, has been demonstrated by some authors, suggesting that they may play complementary roles in the pathogenesis of this fungus on *Bromus tectorum* [28]. Thus, under the objective of developing new-generation herbicides, identifying synergistic allelochemicals may lead to more effective herbicides that require a smaller number of compounds in their composition, thus, minimizing environmental impact [29]. This suggestion regarding the phytotoxic metabolites from *P. grisea* is supported by the strong activity shown by organic extracts obtained at pH 2 from the liquid culture of isolates 5 and 10, where EPYR was not detected (Figure 3C,D). Isolate 1 was also completely suppressive of normal germination at regular pH in the absence of EPYR production, possibly due to another mechanism of action. The activity results presented here denote the direct relationship of the quantity of EPYR with the phytotoxicity of *P. grisea* extracts, as well as the interest in deeper studies on the array of phytotoxic metabolites produced by this fungus and on their potential alternative and synergistic modes of action.

## 4. Conclusions

This study reported, for the first time, a HPLC method for the quantification of (10*S*,11*S*)-(—)-*epi*-pyriculol (EPYR) in a complex matrix. This natural compound is a promising phytotoxin produced by the foliar fungal pathogen *P. grisea*, isolated from the invasive weed species buffelgrass in North America. Considering the effective control of buffelgrass has not yet been achieved, this compound could be a suitable alternative to synthetic pesticides as an active principle of a new biopesticide for buffelgrass management. To further investigate this possibility, the HPLC method was developed to quantify the EPYR in the organic extracts obtained from twelve isolates of *P. grisea* collected from diseased buffelgrass leaves. The results demonstrated that the production of EPYR varied among the isolates and allowed us to select a high-producing strain that could be used for defining the best cultural conditions for its mass production. Larger quantities of EPYR are needed to continue studies on its potential application as a bioherbicide against buffelgrass. In particular, a suitable formulation should be developed to enhance EPYR bioavailability and increase its solubility in water. Natural cyclodextrins could be employed for this purpose, as previously performed with other bioactive compounds. In fact, these cyclic oligosaccharides can encapsulate different hydrophobic metabolites, having one hydrophobic cavity capable of hosting molecules of different sizes and a hydrophilic surface that renders them soluble in aqueous media [30]. This system has been applied in recent studies for the development of new pre-emergent herbicides for weed biocontrol [31]. Another important point for the high-yield production and the practical application of EPYR is the evaluation of its ecotoxicological profile. Finally, the results of this study highlighted the strong relationship between the phytotoxicity of the twelve isolates and the production of EPYR. It could also prompt the investigation of some isolates for the production of other phytotoxic metabolites.

## Figures and Tables

**Table 1:** Data about the twelve isolates of *Pyricularia grisea*, their culture filtrates, and organic extracts ^1^.

Isolate Number	*P. grisea* Isolate	Lyophilized Culture Filtrate Weight (g)	Lyophilized Mycelial Weight (g)	pH of the Culture Filtrate	EtOAc Organic Extract at Regular pH (mg)	EtOAc Organic Extract at pH 2 (mg)
1	CB 5B2	1.73	0.37	8	3.61	7.22
2	CB 5C2	1.36	0.37	8	7.56	15.74
3	MR 4D1	1.57	0.42	9	6.00	10.12
4	SNM 2-1A3	1.67	0.37	8	12.95	14.25
5	SNM 2-2A1	1.84	0.27	5	5.17	21.67
6	SNM 2-2A2	1.69	0.36	8	5.34	14.33
7	SNM 3B2	1.79	0.33	8	5.19	6.75
8	SNM 3E2A	2.12	0.21	5	6.69	27.21
9	SNM 5A2	1.49	0.36	9	16.04	14.40
10	SNM 5B1	2.22	0.20	5	5.23	19.57
11	SNM 5B2	2.04	0.29	7	5.55	12.63
12	SNM 5D1	1.61	0.34	8	5.08	19.80

^1^ The values refer to the isolates grown in 125 mL of PDB.

**Table 2:** Analytical characteristics of the calibration curve ^1^ for the quantification of EPYR by HPLC.

Retention Time (min)	Range (µg)	R^2^	Data Points	Limit of Detection (µg)
17.373	0.0078–5	0.998	20	0.0078

^1^ Calculated with the equation form “y = a + bx”, where the “y” is the peak area and “x” the amount (µg) of EPYR injected. The calibration curve was: y = 8 × 10^6^ x—63,315.

**Table 3:** Relative amount (% *w/w*) of EPYR in the organic extracts of *Pyricularia grisea* isolates (1–12) obtained at regular pH and pH 2 and its production yields (mg/L) in PDB liquid culture calculated with EPYR data amounts using the developed HPLC method.

Isolate Number	*P. grisea* Isolate	Regular pH		pH 2	
		% *w/w* EPYR in the Organic Extract	Production Yield (mg/L) of EPYR ^1^	% *w/w* EPYR in the Organic Extract	Production Yield (mg/L) of EPYR ^1^
1	CB 5B2	0.30	0.09 ± 0.02	0.03	0.02 ± 0.01
2	CB 5C2	0.09	0.05 ± 0.01	0.06	0.07 ± 0.02
3	MR 4D1	3.83	1.83 ± 0.09	0.29	0.23 ± 0.06
4	SNM 2-1A3	3.95	4.07 ± 0.13	1.95	2.22 ± 0.09
5	SNM 2-2A1	0	0	0	0
6	SNM 2-2A2	0.21	0.09 ± 0.02	0.14	0.16 ± 0.02
7	SNM 3B2	0.11	0.04 ± 0.01	0.09	0.05 ± 0.01
8	SNM 3E2A	0	0	0	0
9	SNM 5A2	8.17	10.46 ± 0.21	2.56	2.95 ± 0.08
10	SNM 5B1	0.15	0.06 ± 0.02	0	0
11	SNM 5B2	0.09	0.03 ± 0.01	0.16	0.16 ± 0.01
12	SNM 5D1	0	0	0	0

^1^ Data are mean ± standard error (*n* = 3).

## Data Availability

Not applicable.

## Supplementary Materials

The following supporting information can be downloaded at: https://www.mdpi.com/article/10.3390/jof9030316/s1, S1: ^1^H NMR spectrum and spectroscopic data of (10*S*,11*S*)-(—)-*epi*-pyriculol (CDCl_3_; 400 MHz); S2: ESI MS spectrum of (10*S*,11*S*)-(—)-*epi*-pyriculol recorded in positive mode; S3: graphical representation of the calibration curve of (10*S*,11*S*)-(—)-*epi*-pyriculol standard; S4: chromatograms of the organic extracts obtained at regular pH and pH 2 of isolates 1–12.
