# Supplementary material for: Production of (10S,11S)-(—)-epi-Pyriculol and Its HPLC Quantification in Liquid Cultures of Pyricularia grisea, a Potential Mycoherbicide for the Control of Buffelgrass (Cenchrus ciliaris)
Source: J Fungi (Basel). 2023 Mar 3;9(3):316. doi: 10.3390/jof9030316 (PMC10056936; doi:10.3390/jof9030316)
Supplement: Supplementary file 1 [file jof-09-00316-s001.zip › jof-2239526-supplementary.pdf]

Supplementary Information

Production of (10*S*,11*S*)-(—)-*epi*-Pyriculol and Its HPLC Quantification in Liquid Cultures of *Pyricularia grisea*, a Potential Mycoherbicide for the Control of Buffelgrass (*Cenchrus ciliaris*)

Jesús G. Zorrilla [1,2], Marco Masi [1,*], Suzette Clement [3], Alessio Cimmino [1] and Susan Meyer [3]

[1] Department of Chemical Sciences, University of Naples Federico II, Complesso Universitario Monte S. Angelo, Via Cintia, 80126 Naples, Italy
[2] Allelopathy Group, Department of Organic Chemistry, Facultad de Ciencias, Institute of Biomolecules (INBIO), University of Cadiz, C/Avenida República Saharaui, s/n, 11510 Puerto Real, Spain
[3] Shrub Sciences Laboratory, U.S. Forest Service Rocky Mountain Research Station, 369 N. 100 West Suite 8, Cedar City, UT 84721, USA
* Correspondence: marco.masi@unina.it

S1. ^{1}H NMR spectrum and spectroscopic data of (10*S*,11*S*)-(—)-*epi*-pyriculol ($CDCl_3$; 400 MHz) . 2

S2. ESI MS spectrum of (10*S*,11*S*)-(—)-*epi*-pyriculol recorded in positive mode 3

S3. Graphical representation of the calibration curve of (10*S*,11*S*)-(—)-*epi*-pyriculol standard 3

S4. Chromatograms of the organic extracts obtained at regular pH and pH 2 of isolates 1-12 4

S1. ^{1}H NMR spectrum and spectroscopic data of (10*S*,11*S*)-(—)-*epi*-pyriculol ($CDCl_3$; 400 MHz)

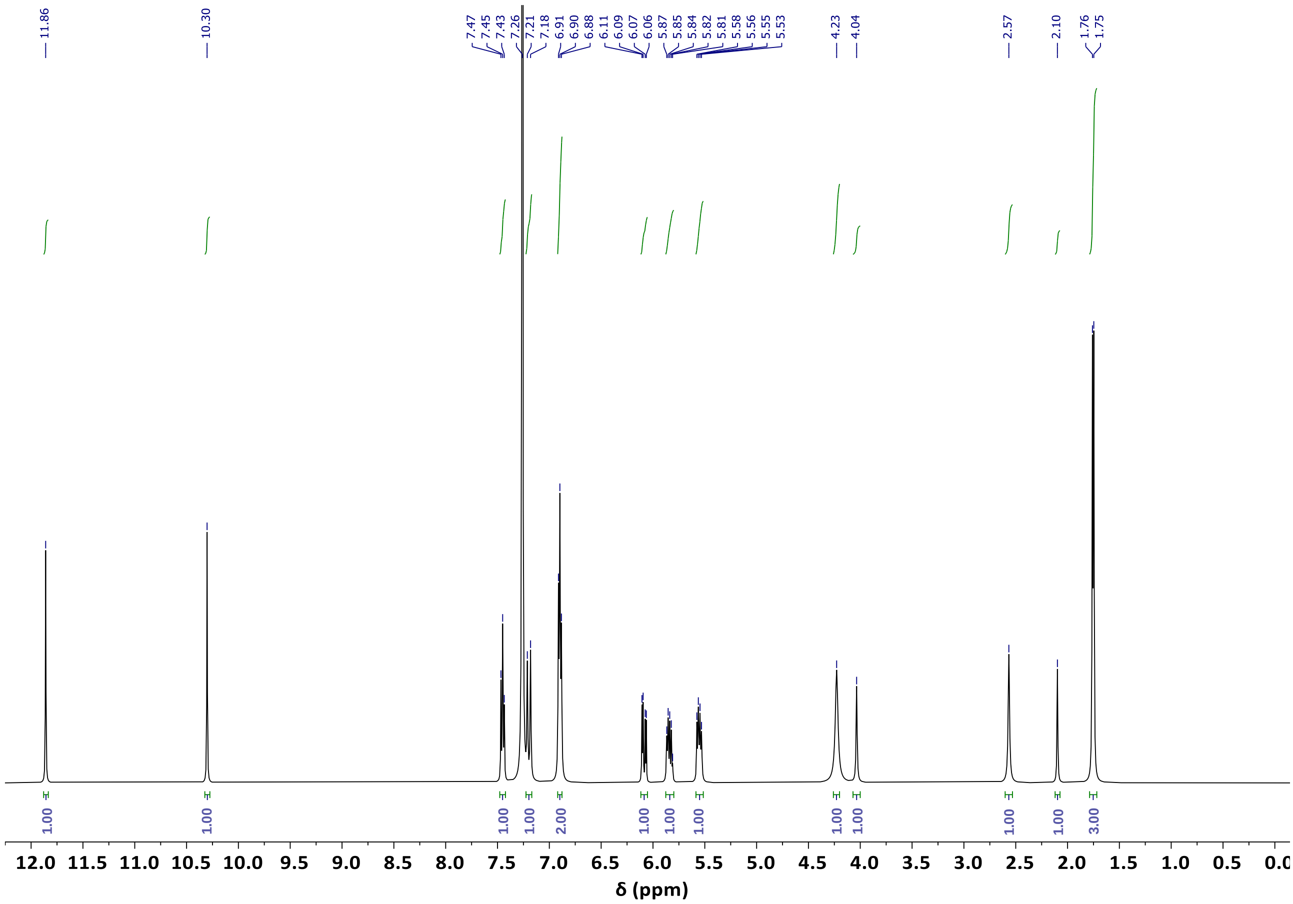

Figure S1. ^{1}H NMR spectrum (400 MHz) of (10*S*,11*S*)-(—)-*epi*-pyriculol in $CDCl_3$

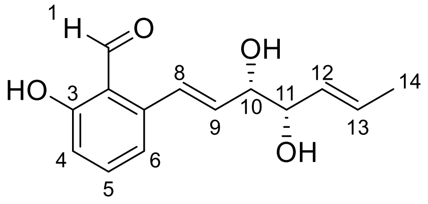

^{1}H NMR ($CDCl_3$, 400 MHz), δ (ppm):

11.86 (1H, s, OH-3); 10.30 (1H, s, H-1); 7.45 (1H, t, *J* = 8.0 Hz, H-5); 7.20 (1H, d, *J* = 15.5 Hz, H-8); 6.90 (2H, m, H-4 and H-6); 6.08 (1H, dd, *J* = 15.5 and 5.4 Hz, H-9); 5.86 (1H, dd, *J* = 14.7 and 6.3 Hz, H-13); 5.56 (1H, dd, *J* = 14.7 and 7.1 Hz, H-12); 4.23 (1H, br s, H-10); 4.04 (1H, br s, H-11); 2.57 (1H, s, OH-10/11); 2.10 (1H, s, OH-10/11); 1.76 (3H, d, *J* = 6.3 Hz, H-14).

S2. ESI MS spectrum of (10*S*,11*S*)-(—)-*epi*-pyriculol recorded in positive mode.

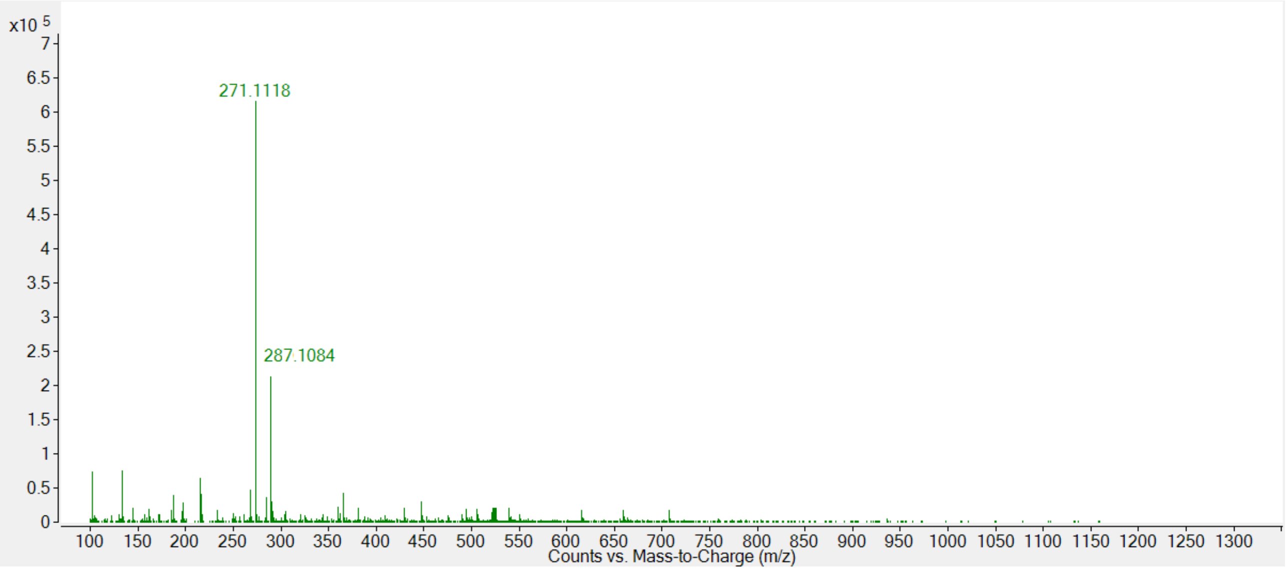

Figure S2. ESI MS spectrum of (10*S*,11*S*)-(—)-*epi*-pyriculol recorded in positive mode.

S3. Graphical representation of the calibration curve of (10*S*,11*S*)-(—)-*epi*-pyriculol standard.

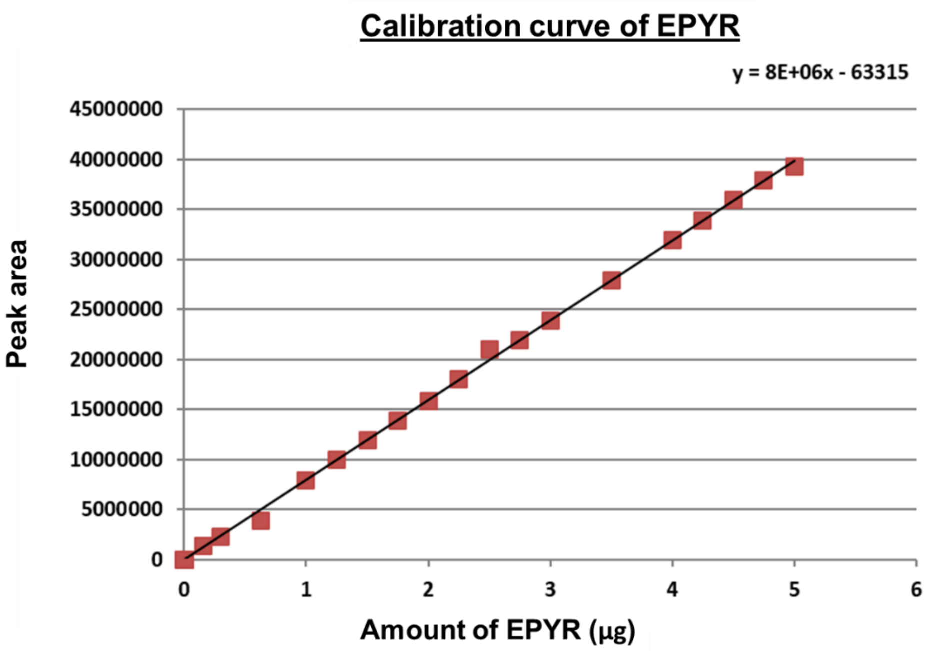

Figure S3. Calibration curve of the (10*S*,11*S*)-(—)-*epi*-pyriculol standard.

S4. Chromatograms of the organic extracts obtained at regular pH and pH 2 of isolates 1-12.

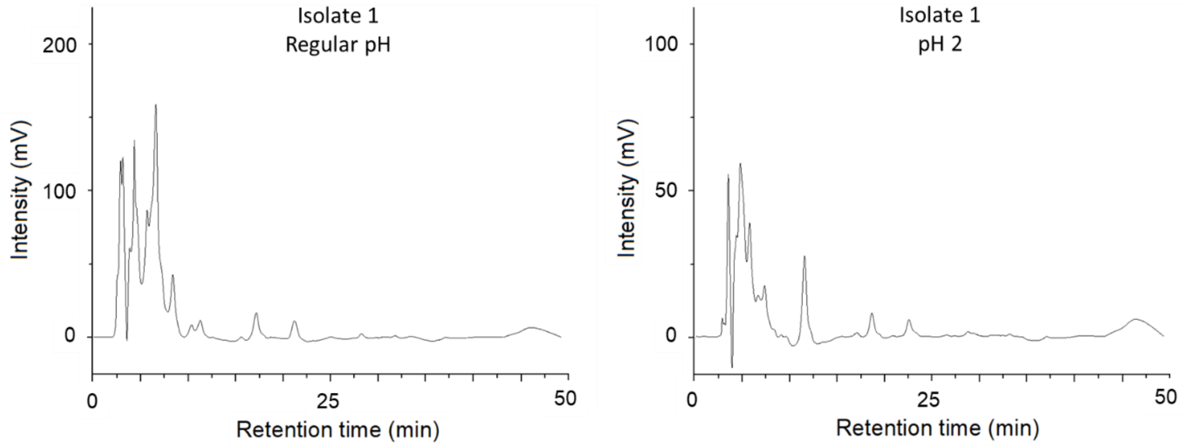

Figure S4. Chromatogram of the organic extract obtained at regular pH (left) and pH 2 (right) of isolate 1 (CB 5B2) analyzed at 2 mg/mL.

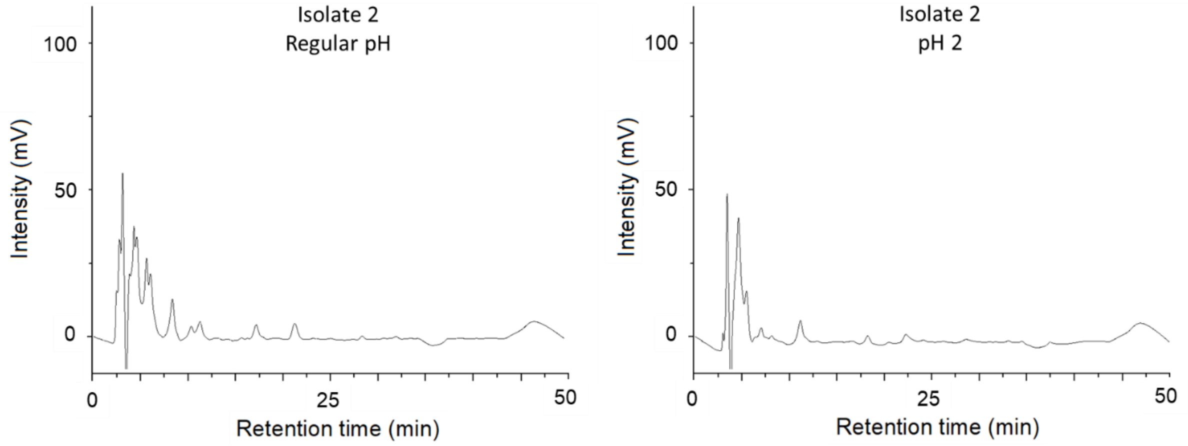

Figure S5. Chromatogram of the organic extract obtained at regular pH (left) and pH 2 (right) of isolate 2 (CB 5C2) analyzed at 2 mg/mL.

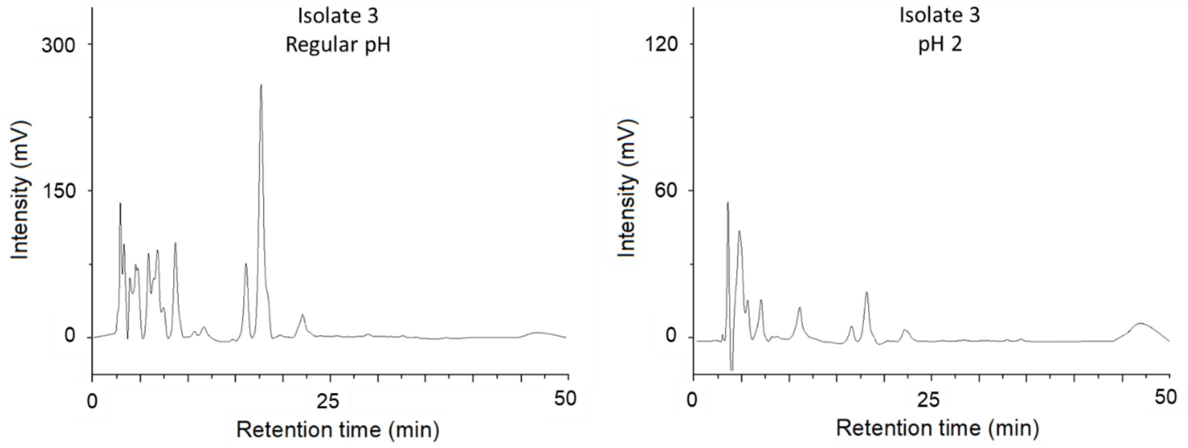

Figure S6. Chromatogram of the organic extract obtained at regular pH (left) and pH 2 (right) of isolate 3 (MR 4D1) analyzed at 2 mg/mL.

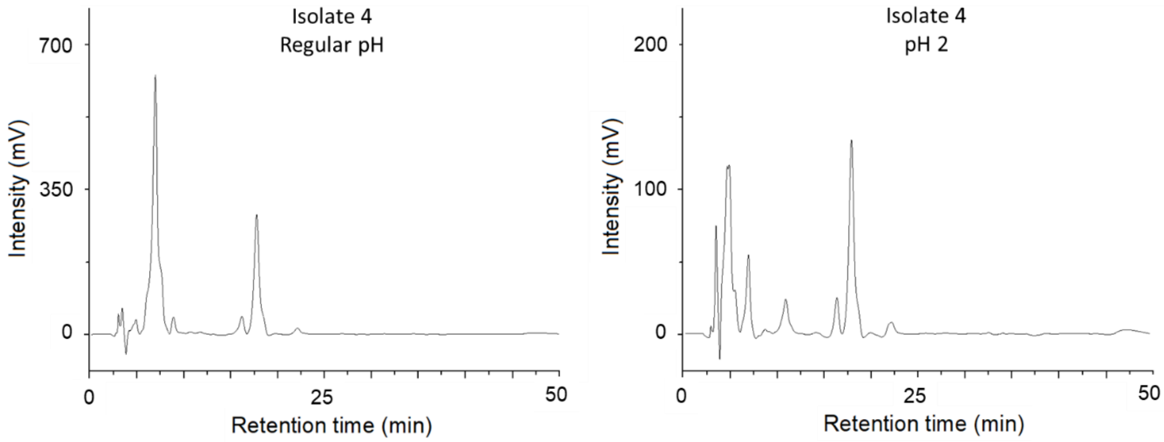

Figure S7. Chromatogram of the organic extract obtained at regular pH (left) and pH 2 (right) of isolate 4 (SNM 2-1A3) analyzed at 2 mg/mL.

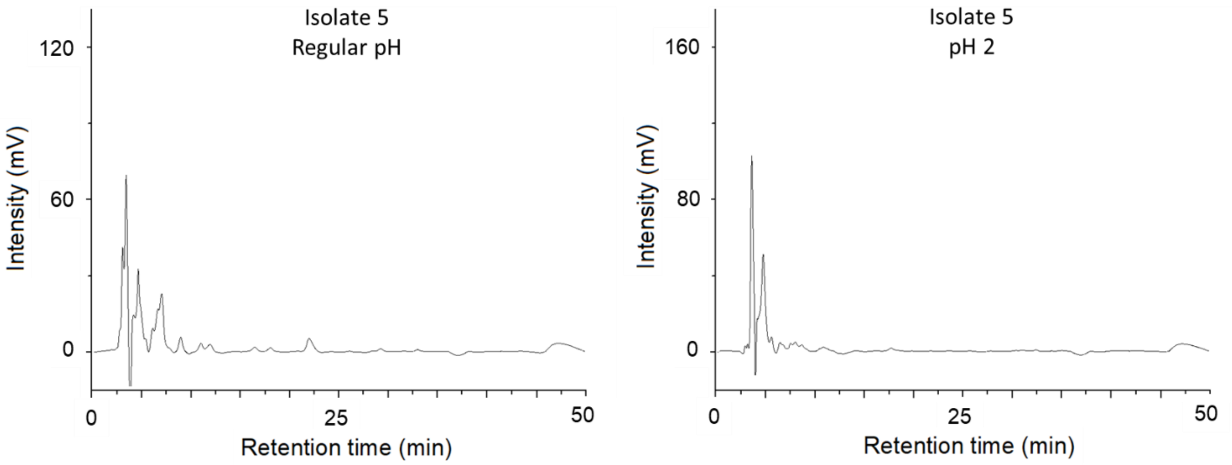

Figure S8. Chromatogram of the organic extract obtained at regular pH (left) and pH 2 (right) of isolate 5 (SNM 2-2A1) analyzed at 2 mg/mL.

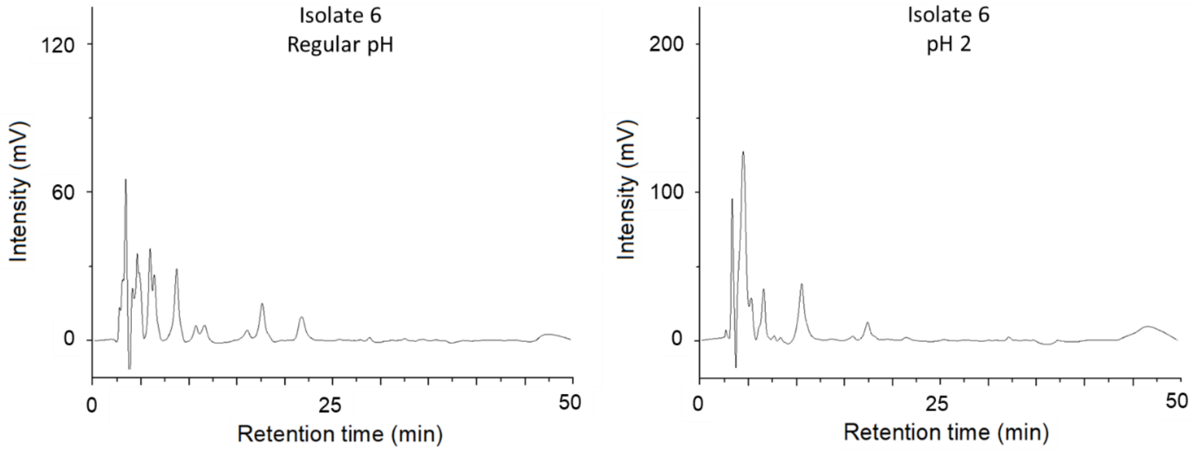

Figure S9. Chromatogram of the organic extract obtained at regular pH (left) and pH 2 (right) of isolate 6 (SNM 2-2A2) analyzed at 2 mg/mL.

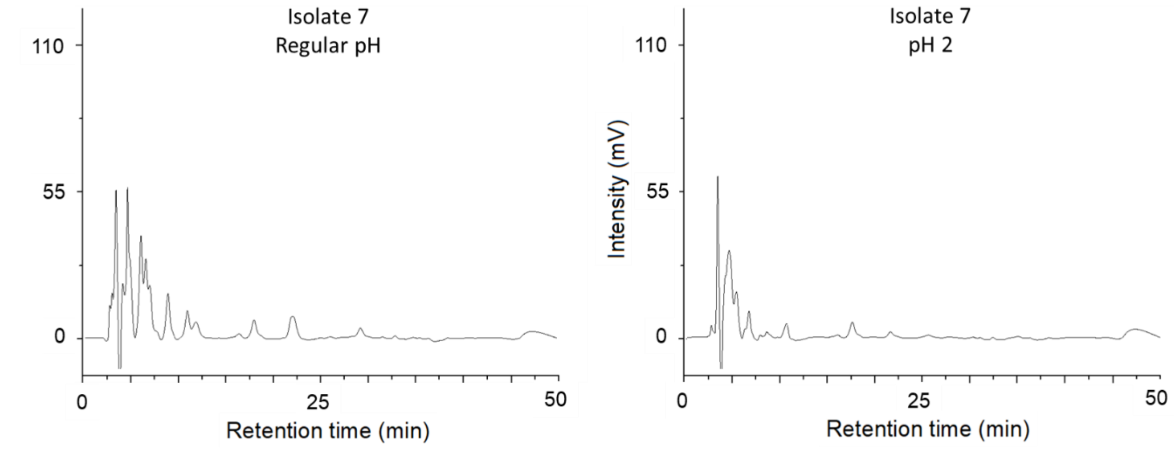

Figure S10. Chromatogram of the organic extract obtained at regular pH (left) and pH 2 (right) of isolate 7 (SNM 3B2) analyzed at 2 mg/mL.

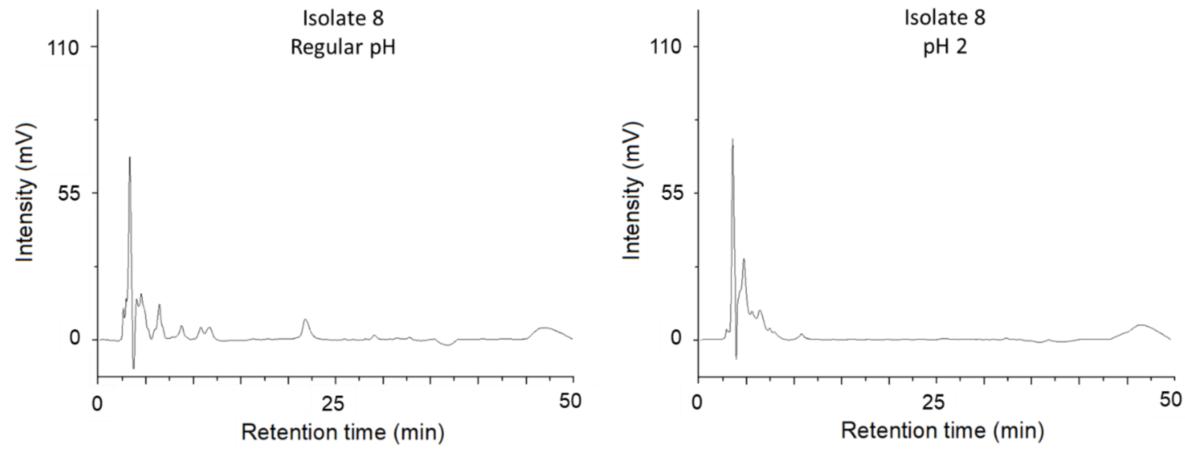

Figure S11. Chromatogram of the organic extract obtained at regular pH (left) and pH 2 (right) of isolate 8 (SNM 3E2A) analyzed at 2 mg/mL.

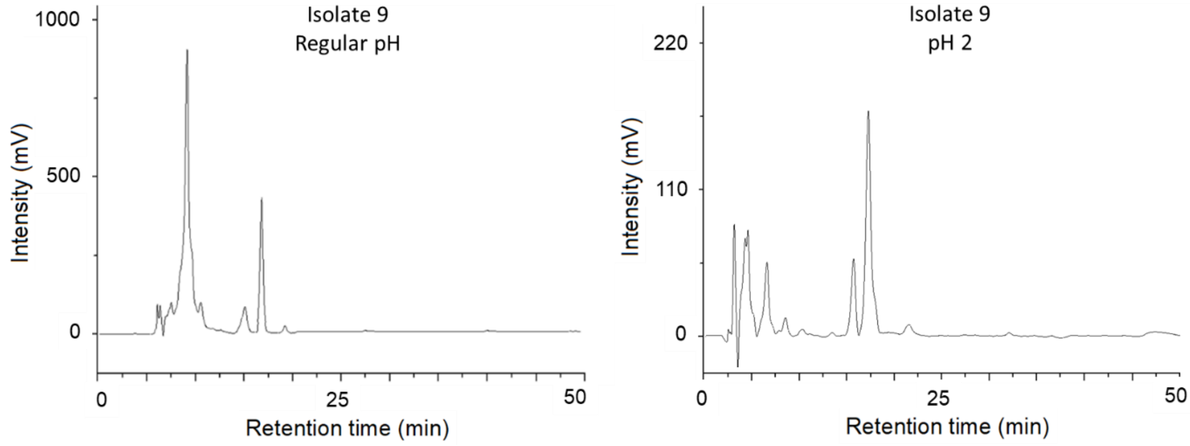

Figure S12. Chromatogram of the organic extract obtained at regular pH (left) and pH 2 (right) of isolate 9 (SNM 5A2) analyzed at 2 mg/mL.

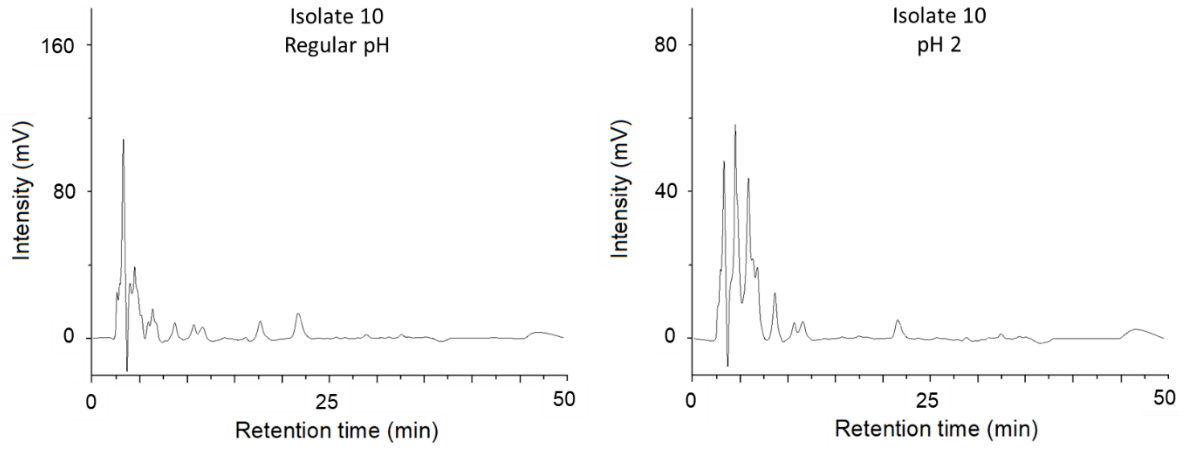

Figure S13. Chromatogram of the organic extract obtained at regular pH (left) and pH 2 (right) of isolate 10 (SNM 5B1) analyzed at 2 mg/mL.

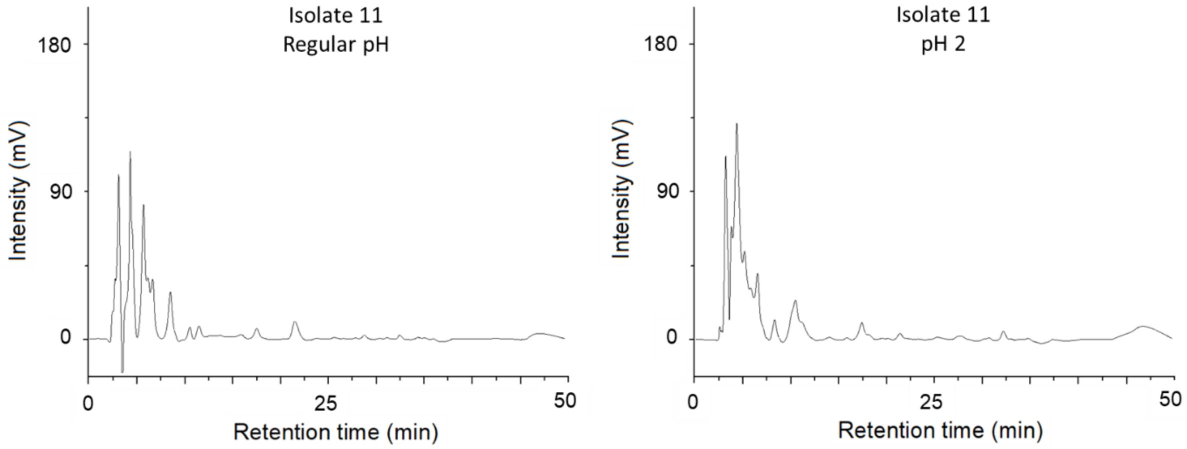

Figure S14. Chromatogram of the organic extract obtained at regular pH (left) and pH 2 (right) of isolate 11 (SNM 5B2) analyzed at 2 mg/mL.

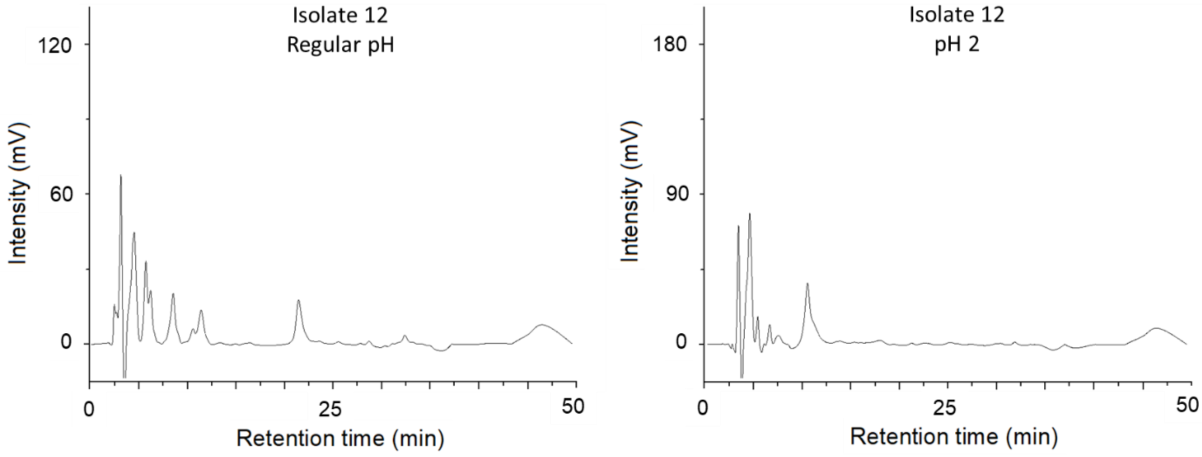

Figure S15. Chromatogram of the organic extract obtained at regular pH (left) and pH 2 (right) of isolate 12 (SNM 5D1) analyzed at 2 mg/mL.
